# Assessment of cardiac radiation dose in the Co-60 prone-based stereotactic partial breast irradiation using distance metrics

**DOI:** 10.3389/fonc.2025.1458111

**Published:** 2025-09-04

**Authors:** Young Suk Kwon, David Parsons, Mona Arbab, Narine Wandrey, Prashant Yarlagadda, Strahinja Stojadinovic, Weiguo Lu, Prasanna Alluri, Xingzhe Li, Tsuicheng Chiu, Muhan Lin, Liyuan Chen, D.W.Nathan Kim, Yesenia Gonzalez, Xuejun Gu, You Zhang, Robert Timmerman, Asal Rahimi

**Affiliations:** ^1^ Department of Radiation Oncology, The University of Texas Southwestern Medical Center, Dallas, TX, United States; ^2^ Department of Radiation Oncology, Vanderbilt University Medical Center, Nashville, TN, United States; ^3^ Department of Radiation Oncology, Stanford University, Palo Alto, CA, United States

**Keywords:** breast cancer, cardiotoxicity, cardiac radiation dose, early-stage breast cancer, partial breast irradiation, radiation therapy

## Abstract

**Purpose/objective(s):**

The GammaPod™ (GP) system, a contemporary platform dedicated to breast cancer (BC) radiotherapy, facilitates the delivery of accelerated partial breast irradiation (APBI) via the Co-60 prone-based stereotactic partial breast irradiation (CP-sPBI) technique. The precise CP-sPBI configuration permits reduced planning target volume (PTV) margins compared to other APBI techniques, creating an increased separation between PTV and organs at risk (OARs). This study explores the variability of heart-to-PTV distance and its effects on cardiac dosimetry.

**Materials/methods:**

An APBI database of 102 consecutive patients treated with CP-sPBI between March 2019 and February 2023 was queried for retrospective analysis. Statistical analyses were performed to evaluate the mean and maximum (max) heart and left anterior descending artery (LAD) doses based on two parameters: 1) D-H, the minimum distance between the heart and the lumpectomy cavity PTV, and 2) D-LAD, the minimum distance between the LAD and the lumpectomy cavity PTV. The median values of D-H and D-LAD, measured on either axial or sagittal planes, were employed to categorize patients based on cardiac dose levels.

**Results:**

The analysis revealed a statistically significant difference in the mean and max heart dose between patients with left-sided and right-sided breast cancer. Specifically, in left-sided breast cancer patients, median D-H and D-LAD cutoffs were identified as 2.67 and 3.22 cm, respectively. Patients with D-H less than 2.67 cm exhibited significantly higher mean (1.77 vs. 0.75 Gy; p < 0.01) and max heart doses (15.21 vs. 4.38 Gy; p < 0.01) compared to those with D-H greater than or equal to 2.67 cm. Similarly, lower D-LAD values (<3.22 cm) demonstrated a statistically significant association with increased arterial dose compared to higher D-LAD values (≥3.22 cm).

**Conclusions:**

Leveraging its sharp dose fall-off characteristic, the GP treatment delivery system facilitates the delivery of five-fraction APBI while maintaining acceptable cardiac dosimetry parameters. This is particularly advantageous for tumors situated further from the heart because heart doses dissipate with distance. The estimates of heart dose based on the distance to the heart and LAD from PTV have the potential to serve as a valuable tool for clinicians, aiding in more refined risk evaluation and patient selection for CP-sPBI.

## Introduction

Breast cancer (BC) is the most common type of cancer in women. Recent advancements in radiation techniques have allowed for more localized and shorter forms of radiation, including accelerated partial breast irradiation (APBI). APBI has led to less acute and chronic radiation-induced toxicities and better cosmetic outcomes as reported by patients and physicians. It has also resulted in similar ipsilateral breast recurrence rates and BC-specific survival rates when compared to those of whole breast irradiation (1).

APBI can be delivered using different radiation techniques. This includes intracavitary brachytherapy, intraoperative radiation therapy (IORT), and external beam radiation therapy (2). The recent data from the TARGIT-IORT have demonstrated the maximum protection of organs at risk while maintaining a long-term oncologic control by the use of IORT at the time of lumpectomy (3). However, the widespread adoption of IORT is limited due to technical procedural expertise (4).

GammaPod™ (GP) (Xcision Medical Systems, LLC, Columbia, MD, USA) is a novel, prone-based breast stereotactic radiosurgery (SRS) system developed at the University of Maryland Medical Center. It utilizes 24 rotating Co-60 sources to generate highly conformal dose distributions for breast treatments. This technology is indicated for tumor cavity boost treatment, partial breast irradiation, pre-surgical SRS, or single-fraction definitive treatment (5, 6). Given its small field sizes and non-standard geometry, it requires modified calibration protocols based on the American Association of Physicists in Medicine Task Group 21 (TG-21) and the International Atomic Energy Agency (IAEA) Technical Report Series 483 (TRS 483) for reference dose measurements (5, 6). The radiation delivery is accurate due to the presence of a vacuum-assisted breast immobilization cup (7). This has allowed for smaller planning target volume (PTV) margins, and our prior study has demonstrated that using 3 mm from clinical target volume (CTV) to generate PTV is reproducible (6).

One of the major long-term radiation toxicity concerns is the increased risk of coronary artery events due to radiation. For conventional radiation therapy breast treatments, there is a linear increase in the major coronary events by 7.4% per 1-Gy increase in the mean heart dose. This increase can happen within the first 5 years after radiotherapy (RT) and can continue into the third decade after RT (8). Similarly, early-stage Hodgkin’s lymphoma patients treated with PET-directed therapy in a UK randomized trial demonstrated higher rates of radiation-related mortality from cardiac diseases with increased dose levels. For example, the mean predicted 30-year absolute excess risk of radiation-related mortality from heart disease ranged from 0.03% for those receiving <0.5 Gy to 2.20% for those receiving 10+ Gy (9).

Another study by Zureick et al. has examined the radiation dose to cardiac substructure and has shown that the mean and maximum (max) left anterior descending artery (LAD) doses, in addition to the mean heart dose, could predict increased risk of cardiac disease (10). A mean LAD dose exceeding 2.8 Gy is associated with an increased risk of any cardiac event. Therefore, it is important to understand factors that could influence dose gradients and the subsequent mean heart dose.

In this study, we seek to evaluate the distance between the heart and PTV as a potential surrogate that can predict the mean and max heart and LAD doses. This would help us risk-stratify patients and prioritize those who may benefit from receiving radiation on the GP machine. Furthermore, our study may aid in optimizing clinical decision-making when selecting a machine among several different technologies that could be utilized for a stereotactic partial breast irradiation (sPBI) program, including GP, CyberKnife™, and MRI- and Cone Beam Computed Tomography (CBCT)-guided adaptive Linear accelerator (LINACs).

## Materials and methods

An sPBI database of 102 consecutive patients treated with Co-60 prone-based sPBI (CP-sPBI) from March 2019 to February 2023 was queried for retrospective analysis at our institution. This study was approved by the The University of Texas, Southwestern Medical Center (UTSW) institutional review board, and all 102 patients provided written informed consent prior to receiving radiotherapy (RT). All patients underwent replanning to a standard sPBI regimen of 30 Gy in five fractions. The CTV was delineated as a 1-cm uniform expansion of the gross tumor volume (GTV), encompassing surgical clips, with contouring adjustments to avoid the chest wall and to maintain a minimum 5-mm clearance from the skin surface. The planning target volume (PTV) was generated by a 0.3-cm uniform expansion of the CTV, ensuring a minimum 5-mm clearance from the skin surface. Organs at risk (OARs) included the skin, heart, LAD, ipsilateral breast, and chest wall, planned in our previously published constraints (11).

**Figure 1:**
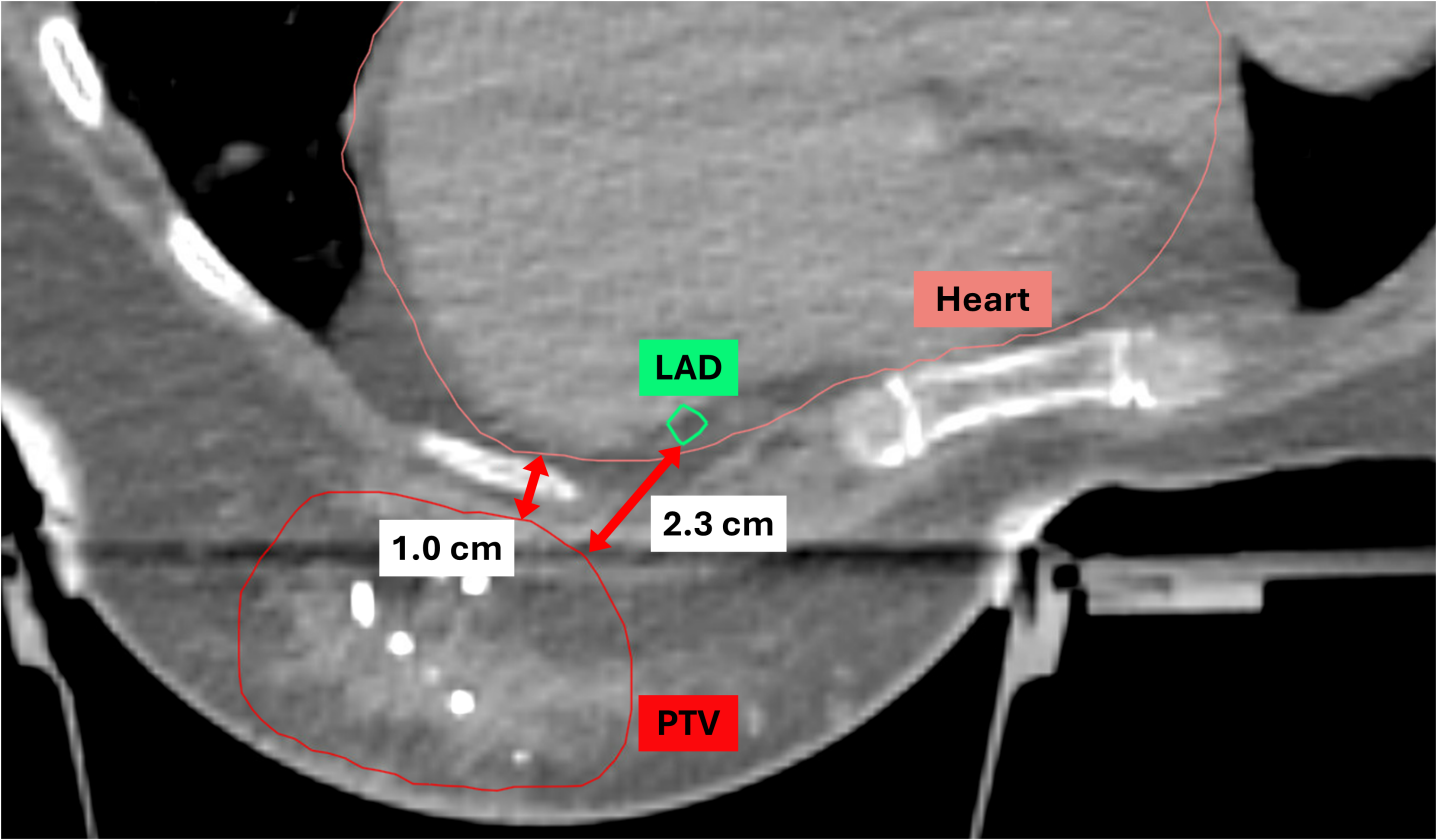
Definitions of D-H and D-LAD. D-H, the minimum distance from the heart to the lumpectomy PTV; D-LAD, the minimum distance from the LAD to the lumpectomy PTV; PTV, planning target volume; LAD, left anterior descending artery.

Statistical analyses were performed to evaluate the mean and maximum heart and LAD doses based on two parameters: 1) D-H, the minimum distance between the heart and the lumpectomy cavity PTV, and 2) D-LAD, the minimum distance between the LAD and the lumpectomy cavity PTV, using the median value cutoff (12). Both D-H and D-LAD values were measured in either the axial or sagittal views (**Figure 1**). The scatterplots describing the relationships between 1) the heart and the D-H and 2) the LAD max and the D-LAD were fitted to inverse exponential functions to obtain the coefficients of determination. Statistical analyses were performed using MATLAB 2023b to assess group differences in the mean values. An independent-samples Student’s t-test was employed to compare the means between the two groups.

## Results

**Figure 2:**
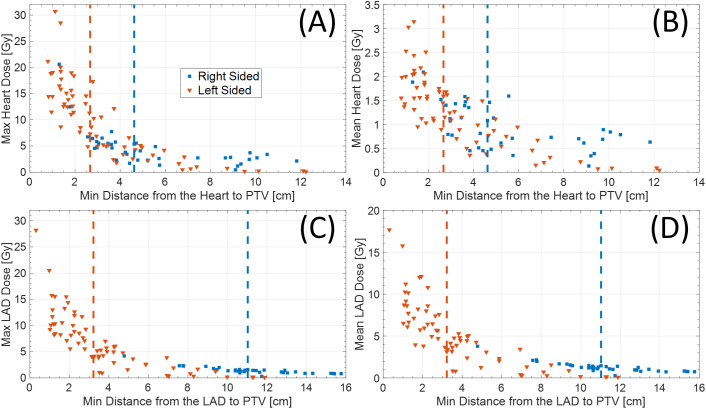
**(A)** Max and **(B)** mean heart dose based on distance from heart to PTV. Similarly, **(C)** max and **(D)** mean LAD dose based on distance from LAD to PTV. Red and blue dotted vertical lines represent median cutoff points for left- and right-sided diseases, respectively. PTV, planning target volume; LAD, left anterior descending artery.

**Figure 3:**
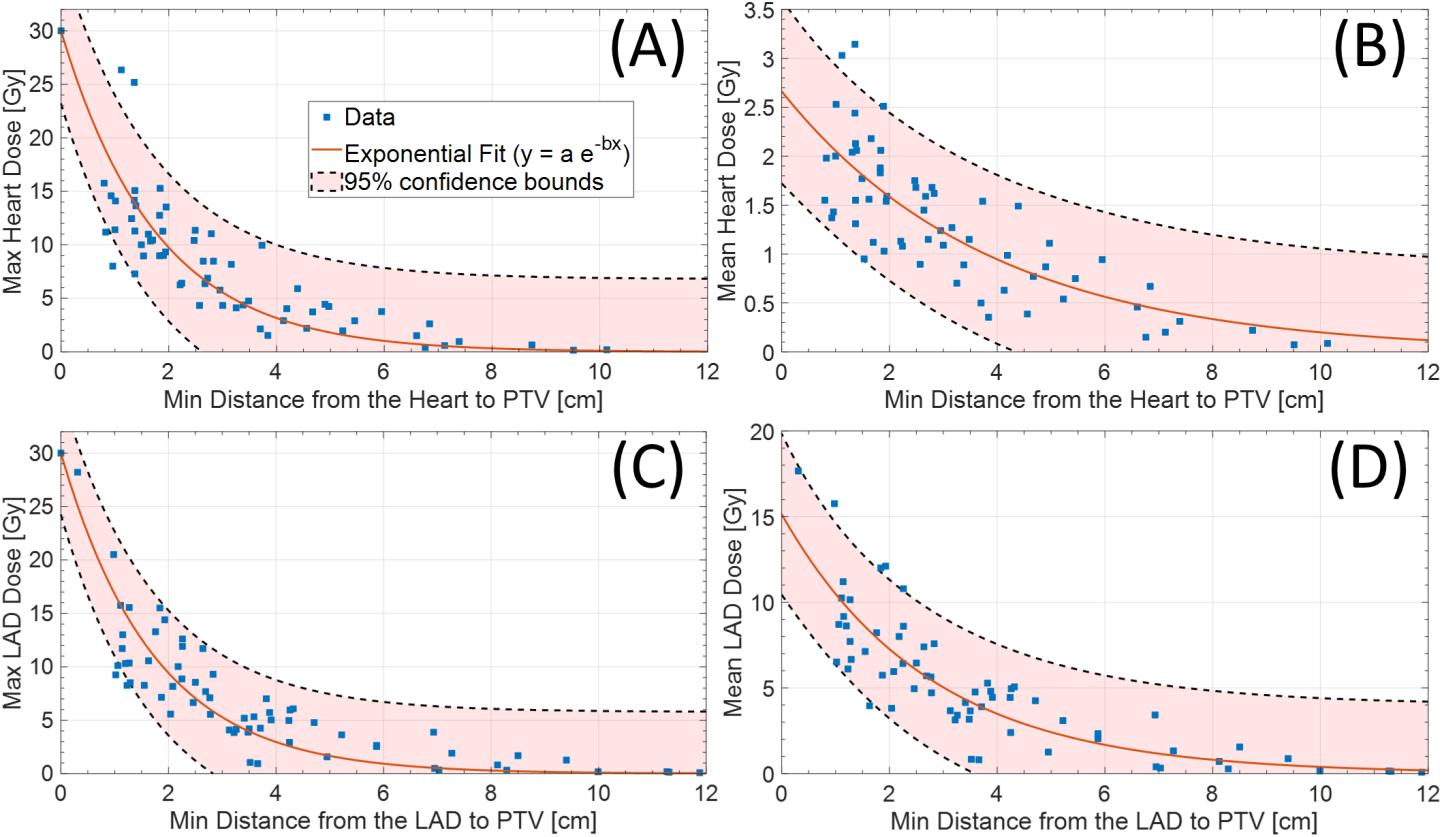
**(A)** Max (adjR^2^ = 0.98) and **(B)** mean (adjR^2^ = 0.67) heart dose based on distance from heart to PTV. Similarly, **(C)** max (adjR^2^ = 0.98) and **(D)** mean (adjR^2^ = 0.72) LAD dose based on distance from LAD to PTV (left-sided only). LAD, left anterior descending artery; PTV, planning target volume.

Among 102 study participants, there were 65 (63.7%) left-sided BC cases and 36 (35.3%) right-sided cases. Among the 102 patients who underwent accelerated partial breast irradiation using GP, none developed adverse events, including cardiac or pulmonary complications, during or after treatment. The mean and max heart doses for patients with left-sided BC were significantly different from the mean heart dose for those with right-sided BC (**Tables 1**, **2**, **Figure 2**). Using a median cutoff of 2.67 cm for the D-H in patients with left-sided BC, the differences between D-H < 2.67 cm and D-H ≥ 2.67 cm for cardiac dosimetric evaluations were all statistically significant, including the mean (1.77 vs. 0.75 Gy; p < 0.01) and max heart doses (15.21 vs. 4.38 Gy; p < 0.01) (**Table 1**). Similarly, using a median cutoff of 3.22 for the D-LAD in patients with left-sided BC demonstrated statistically significant differences between D-LAD < 3.22 cm and D-LAD ≥ 3.22 cm (**Table 2**). The fitted inverse exponential functions demonstrated very high inverse correlations with the adjusted R^2^ of 0.98 for both relationships between the max heart dose and D-H and the max LAD dose and D-LAD (**Figure 3**).

**Table 1:** Heart and LAD dose for the right side.

Heart	D-H < 4.62, mean (range)	D-H ≥ 4.62, mean (range)	p-Value
Max (Gy)	6.31 (1.71–20.6)	2.72 (0.46–5.55)	<0.01
Mean (Gy)	1.18 (0.45–2.09)	0.73 (0.13–1.5)	<0.01

LAD	D-LAD < 11.03, mean (range)	D-LAD ≥ 11.03, mean (range)	p-Value
Max (Gy)	4.86 (1.27–13.32)	2.67 (0.40–4.97)	<0.01
Mean (Gy)	1.59 (0.87–3.78)	0.89 (0.27–1.39)	<0.01

LAD, left anterior descending artery; PTV, planning target volume; D-H, the minimum distance from the heart to the lumpectomy PTV; D-LAD, the minimum distance from the LAD to the lumpectomy PTV.

**Table 2:** Heart and LAD dose for the left side.

Heart	D-H < 2.67, mean (range)	D-H ≥ 2.67, mean (range)	p-Value
Max (Gy)	15.21 (4.88–30.7)	4.38 (0.10–17.30)	<0.01
Mean (Gy)	1.77 (0.90–3.14)	0.75 (0.05–1.68)	<0.01

**LAD**	**D-LAD < 3.22, mean (range)**	**D-LAD ≥ 3.22, mean (range)**	**p-Value**
Max (Gy)	11.41 (4.34–26.35)	4.10 (0.09–14.18)	<0.01
Mean (Gy)	8.04 (3.67–17.67)	2.45 (0.08–5.28)	<0.01

LAD, left anterior descending artery; PTV, planning target volume; D-H, the minimum distance from the heart to the lumpectomy PTV; D-LAD, the minimum distance from the LAD to the lumpectomy PTV.

## Discussion

Our study on cardiac radiation dose in the setting of CP-sPBI demonstrates profound variations in cardiac parameters based on the distance of the target volume to the heart/LAD. Utilizing two different methods of measuring the distance—1) heart to the PTV and 2) LAD to the PTV—we were able to highlight how patient anatomical characteristics and the location of the tumor may influence cardiac dose. While no definitive cardiac threshold values can be drawn from our study, our study findings showed inverse relationships between the doses to the heart/LAD and distances from the heart/LAD to the target volume. This was further illustrated by the decreasing max cardiac doses with distance (adjR^2^ = 0.98 for both max heart and LAD doses) (**Figure 3**).

The etiology of cardiotoxicity associated with radiation is not well-understood and may be a result of several proposed mechanisms. Oxidative stress from radiation leads to an increase in TGF-β1 levels, which can cause endothelial dysfunction and subsequent vascular injury (13). This may manifest in the acute setting as pericarditis or cardiac conduction abnormalities, while late toxicities may emerge years to decades later, as seen with coronary artery disease and valvular dysfunction. One study found that patients with left-sided breast cancer who underwent breast conservation surgery followed by adjuvant radiation had a 6.4% cumulative risk of cardiac-related deaths compared to 3.6% for those with right-sided breast cancer, suggesting that radiation-induced cardiac damage is closely associated with mortality (14). More specifically, it is estimated that the rate of major coronary events increases linearly by 7.4% per gray increase in the mean heart dose (3). Given that higher radiation dose to the heart may accelerate the development of acute and chronic cardiac-related injuries, developing strategies to mitigate doses to the heart will prove to be important, especially for early-stage breast cancer patients whose survival outcomes are minimally affected by the disease process alone.

While there are no definitively established radiation dose constraints for the heart and its substructures, there are several frequently used guidelines. For example, the Quantitative Analysis of Normal Tissue Effects in the Clinic (QUANTEC) estimates less than 1% probability of long-term cardiac mortality with a heart V_25Gy_ less than 10% for conventionally fractionated regimens (15). Other heart constraints for conventional fractionation supported by the QUANTEC include V_30Gy_ less than 46% and a mean heart dose less than 26 Gy. In addition, the Timmerman tables, a gold standard for hypofractionated RT treatment planning, primarily use volume-based constraints for the heart, which vary according to fractionation schedule (16). For five fractions, less than 15 cc of the heart should receive no more than 32 Gy with a max point dose under 38 Gy. Although both dose constraints provide numerical thresholds for heart dose in general, they do not specify which anatomical substructures of the heart are most vulnerable to radiation-associated toxicity.

It is shown that dose to the LAD is associated with cardiac events after RT for BC (10). In this retrospective study of 375 patients with left-sided breast or chest wall irradiation with or without regional nodal irradiation, the authors were able to determine the threshold LAD D_Mean_ Equivalent Dose in 2Gy Fractions (EQD2) of 2.8 Gy using Receiver Operating Characteristic (ROC) curve analysis. While the scope of our study is limited due to the absence of clinical outcomes in the context of early-stage BC, it is evident that patients with left-sided BC with a tumor located close to the LAD had higher doses to the LAD. More specifically, cases with mean LAD > 10 Gy or mean LAD > 15 Gy were all patients with left-sided BC whose distances from the PTV to the LAD were less than 5 cm (**Figure 3**).

In the review of landmark APBI early-stage breast cancer studies, the overall survival outcomes for those treated with five-fraction RT were excellent. The authors of the Florence trial reported that the patients treated with APBI had a similar ipsilateral breast tumor recurrence when compared to those treated with whole breast irradiation (WBI) (17). The cardiac constraints for the APBI arm were V_3Gy_ less than 10%, with a mean dose of 2.5 Gy to 10% of the heart (range 0.0–6.4 Gy). As expected with early-stage BC with a relatively small dose of irradiation in the heart, there were no cardiac-related events specifically discussed in their long-term follow-up.

Similarly, the Formenti single-arm trial evaluated patients with early-stage breast cancer treated with APBI in the prone position utilizing 3D conformal radiation therapy, with the prescription dose of 30 Gy delivered over five fractions over 10 days (18). The only cardiac dose constraint was that the volume of the heart in the treatment field should be less than 10%. After a median follow-up of 64 months, there was one reported death due to heart failure whose condition may not be solely attributed to RT.

While these cardiac parameters in the prior APBI trials are informative, they do not apply to our case of CP-sPBI because of the difference in margin size to PTV and dosimetry due to prone suction cup-based planning. Based on our institutional phase I study on single-fraction stereotactic partial breast irradiation for early-stage BC, our five-fraction SPBI heart constraint is set to be that the percent volume of the heart receiving 5% of the prescription dose should be less than 5% (19). However, this also does not take into account specific arteries in the heart, such as the LAD, and the potential dose impact on cardiac substructures.

To our best knowledge, this is the first study that evaluated the relationship between the distance to the heart and LAD and the mean/max heart and LAD doses in our CP-sPBI delivery system. Based on this study, caution should be exercised with individuals undergoing sPBI when the tumor is closer in proximity to the heart and/or the LAD, particularly those with left-sided BC. More specifically, the inverse relationships between the max heart/LAD doses and the distances to the heart/LAD were evident (adjR^2^ = 0.98) (**Figure 3**). With this information, clinicians may evaluate the minimum distance from the heart and LAD to the estimated PTV region in the breast in advance at the time of the planning scan (cavity check) to better select eligible patients in the prone position on GP (9). We believe that this routine measurement of the minimum distance will facilitate risk stratification in terms of expected cardiac dose and better patient counseling, particularly for those with pre-existing cardiac conditions.

Our institution employs a multi-platform approach to sPBI, utilizing GP, CyberKnife™, and adaptive MR- and CBCT-guided LINAC technologies. In our preliminary data, there were no statistically significant advantages of GP compared to other platforms—including both CT- and MR-based systems—in terms of dose fall-off, conformity, and organ-at-risk sparing (20). Further research is needed to identify patients who may benefit the most from the prone-based CP-sPBI approach. In our current clinical practice, we routinely exclude patients with very close cardiac distance measurements (D-H or D-LAD <1 cm) from treatment with GP. Although we do not yet have validated threshold values, we envision developing a nomogram based on a weighted average model that incorporates cardiac parameters, age, and baseline cardiopulmonary function.

In the near future, we plan to generate comparative dosimetric models to optimize treatment modality selection. These models provide estimates of cardiac exposure for each platform, incorporating patient-specific anatomical data and tumor localization. While clinical evaluation and patient history may guide treatment decisions in select cases, a comprehensive dosimetric assessment often necessitates multiple simulation scans, each configured for a specific treatment modality. Furthermore, we are developing an advanced, artificial intelligence-driven prediction model. This model leverages the anatomical parameters detailed in this manuscript and will be incorporated into a comprehensive training dataset. This dataset will serve as the foundation for a multi-task deep learning model designed to predict the optimal cardiac dose profile achievable across our available treatment platforms (19). The implementation of this predictive modeling framework will facilitate a quantitative, evidence-based approach to treatment modality selection. By prospectively evaluating potential cardiac dosimetry, we aim to minimize treatment-related toxicities and ultimately improve patient outcomes and satisfaction.

### Limitations

This study has inherent limitations associated with its retrospective design, single-institution experience, and lack of long-term clinical follow-up data in assessing the incidence of coronary artery disease (CAD) in the studied population. Our investigation acknowledges the multifactorial nature of CAD development, recognizing that radiation exposure is only one potential contributing factor. Furthermore, the present study focuses on establishing a methodology for cardiac risk assessment for patients undergoing sPBI using GP and, therefore, does not aim to define a definitive dose constraint threshold for the heart and LAD. While existing dosimetric constraints for the heart generally disregard the potential impact on specific cardiac substructures, including the LAD, the optimal parameters remain elusive and cannot be determined based on our current study.

## Conclusion

Limited data currently exist regarding optimal cardiac dose constraints in the context of sPBI. The GammaPod™ system leverages its sharp dose fall-off characteristic to achieve favorable sPBI cardiac dosimetry parameters, particularly for tumors situated further from the heart. The estimates of heart dose based on the distance to the heart and the LAD have the potential to emerge as a valuable clinical tool, enabling more nuanced risk stratification and optimized patient selection for sPBI delivered using the GP system.

## Data Availability

The raw data supporting the conclusions of this article will be made available by the authors, without undue reservation.
